# Developmental Programming in Response to Intrauterine Growth Restriction Impairs Myoblast Function and Skeletal Muscle Metabolism

**DOI:** 10.1155/2012/631038

**Published:** 2012-07-31

**Authors:** D. T. Yates, A. R. Macko, M. Nearing, X. Chen, R. P. Rhoads, S. W. Limesand

**Affiliations:** ^1^Department of Animal Sciences, The University of Arizona, Tucson, AZ 85721-0038, USA; ^2^Department of Animal and Poultry Sciences, Virginia Tech, Blacksburg, VA 24061, USA; ^3^Agricultural Research Complex, Department of Animal Sciences, The University of Arizona, 1650 E. Limberlost Dr., Tucson, AZ 85719, USA

## Abstract

Fetal adaptations to placental insufficiency alter postnatal metabolic homeostasis in skeletal muscle by reducing glucose oxidation rates, impairing insulin action, and lowering the proportion of oxidative fibers. In animal models of intrauterine growth restriction (IUGR), skeletal muscle fibers have less myonuclei at birth. This means that myoblasts, the sole source for myonuclei accumulation in fibers, are compromised. Fetal hypoglycemia and hypoxemia are complications that result from placental insufficiency. Hypoxemia elevates circulating catecholamines, and chronic hypercatecholaminemia has been shown to reduce fetal muscle development and growth. We have found evidence for adaptations in adrenergic receptor expression profiles in myoblasts and skeletal muscle of IUGR sheep fetuses with placental insufficiency. The relationship of **β**-adrenergic receptors shifts in IUGR fetuses because Adr**β**2 expression levels decline and Adr**β**1 expression levels are unaffected in myofibers and increased in myoblasts. This adaptive response would suppress insulin signaling, myoblast incorporation, fiber hypertrophy, and glucose oxidation. Furthermore, this **β**-adrenergic receptor expression profile persists for at least the first month in IUGR lambs and lowers their fatty acid mobilization. Developmental programming of skeletal muscle adrenergic receptors partially explains metabolic and endocrine differences in IUGR offspring, and the impact on metabolism may result in differential nutrient utilization.

## 1. Introduction 

Intrauterine growth restriction (IUGR) affects 10–15% of all infants born in the USA and as many as 24% of babies born in developing countries [1, 2]. Worldwide, IUGR is the second leading cause of perinatal morbidity and mortality behind premature birth [3] and is a major predisposing factor to metabolic disorders throughout postnatal life [4, 5]. Children born SGA due to IUGR are more likely to develop insulin resistance and obesity at young ages [6–8]. As adults, these individuals face greater incidence of type 2 diabetes, hypertension, and other health issues [9–12]. In fact, IUGR offspring are 18 times more likely to develop metabolic syndrome than offspring born at an appropriate size for their gestational age (AGA) [10, 13]. Preterm infants may also be predisposed to metabolic disorders later in life. Though AGA at birth, these infants are often growth-restricted between birth and term because their oral intake of protein cannot match the levels supplied by the placenta [14]. Skeletal muscle accounts for ~40% of the body's mass and thus plays a major role in metabolic homeostasis. Growth and metabolism of skeletal muscle are influenced by a number of factors, including nutrient availability, growth factors, and endocrine signals. In this paper, we will focus on the role of the adrenergic system in fetal adaptations to intrauterine insults alter growth, development, and metabolic set-points in skeletal muscle during late gestation and throughout postnatal life.

## 2. IUGR Conditions: Hypoxemia, Hypoglycemia, and Hypercatecholaminemia

A frequent cause of IUGR is placental insufficiency [15], which can occur spontaneously and from undiagnosed etiology. As the fetus grows, the stunted placenta cannot keep up with the increasing nutritional demands of the fetus, resulting in chronic fetal hypoglycemia and hypoxemia throughout late gestation. These conditions elevate circulating catecholamine concentrations [16]. Plasma norepinephrine and epinephrine concentrations are modestly elevated by fetal hypoglycemia [17–19] but greatly elevated by hypoxemia [20, 21]. Fetal adrenal chromaffin cells contain oxygen-sensitive K^+^ channels that stimulate catecholamine secretion in response to low blood oxygen content, while the splanchnic nerve develops [21, 22]. In IUGR human and rat fetuses, hypoxemia increases catecholamine concentrations in plasma and amniotic fluid by as much as 5-fold [23–25]. Plasma epinephrine and norepinephrine are also elevated in IUGR fetal sheep where placental insufficiency is the known etiology [26–28]. Catecholamines act via the G-protein coupled receptors, Adr*α* and Adr*β* [29, 30], which express multiple subtypes (*α*1A, *α*1B, *α*1D, *α*2A, *α*2B, *α*2C, *β*1, *β*2, and *β*3) with distinct physiological and pharmacological properties [31]. Receptor expression patterns determine how tissues respond to catecholamines, and skeletal muscle predominantly expresses Adr*β*1 and Adr*β*2 subtypes, but Adr*β*3 and Adr*α* subtypes are also present. Even in healthy pregnancies, brief cord occlusions and Poseiro effects cause transient periods of fetal hypoxemia and hypoglycemia [32, 33], making it necessary for the fetus to have a protective mechanism to conserve glucose and oxygen. Skeletal muscle accounts for ~65% of fetal glucose consumption and its metabolic functions are responsive to endocrine regulation [34], making it a prime site for glucose and oxygen conservation.

## 3. Fetal Adaptive Response to IUGR Conditions

Both hypoxemia and hypoglycemia can impact global fetal metabolism, and the response depends upon the duration of the insult. We have shown that acute (<1 hour) fetal hypoxemia suppresses glucose-stimulated insulin secretion by increasing circulating norepinephrine and epinephrine (Yates and Limesand, unpublished), which then activate inhibitory Adr*α*2 receptors on pancreatic *β*-cells [20, 26, 35, 36]. The combination of high circulating catecholamines and low insulin concentrations contributes to hyperlactatemia, acidosis, and hypocarbia in the fetus [37] (Yates and Limesand, unpublished). We postulate that this reflects a temporary reduction in skeletal muscle glucose oxidation to spare glucose and oxygen for neural tissues. This transient coping mechanism is accompanied by increased utilization of nonglucose substrates for energy production. To illustrate, skeletal muscle enzymes associated with fatty acid oxidation are upregulated in fetal rats 24 hours after uterine artery ligation [38], and fatty acid mobilization rates in the sheep fetus increase after six hours of hypoglycemia [17]. Additionally, a greater proportion of amino acids are diverted for oxidization in these fetal sheep [39, 40]. Placental insufficiency causes a chronic state of fetal hypoxemia and hypoglycemia, and therefore hypercatecholaminemia and suppression of glucose oxidation are sustained. As a result, endocrine and metabolic adaptations develop to conserve fetal nutrients by lowering skeletal muscle energy requirements for protein synthesis and growth [41–43]. Accordingly, amino acid oxidation rates in the fetal sheep return to normal after the 8th week of hypoglycemia [41]. Similarly, the ability to mobilize fatty acids is reduced in the IUGR sheep fetus near term [44–46]. In addition to lower oxidative metabolism, the IUGR fetus induces hepatic glucose production and the Cori cycle [47], which utilizes lactate produced by anaerobic glycolysis in skeletal muscle as a substrate for glucose [47, 48]. Lactate clearance by the liver stabilizes plasma lactate concentrations in IUGR fetuses, creating only mild hyperlactatemia [47] compared to acutely hypoxemic fetuses. Thus, long durations of nutrient or oxygen deprivation produce a metabolic shift that may be explained by adaptations to catecholamine levels in fetal circulation.

Comparisons between fetal sheep made chronically hypoglycemic and those with placental insufficiency (hypoxemic and hypoglycemic) show that hypoxemia has a greater propensity than hypoglycemia for inducing metabolic adaptations, possibly due to greater adrenergic activity associated with hypoxemia. Chronic hypoglycemia increases protein breakdown and rates of amino acid oxidation, lowers plasma insulin and glucose uptake, and slows fetal growth rate, but the response is transient and euglycemic recovery normalizes these parameters within a few days [39, 49]. Conversely, in fetal sheep with placental insufficiency, euglycemic correction fails to restore glucose homeostasis or improve growth rate and in fact worsens hypoxemia and hypoinsulinemia, resulting in acidosis [50]. Therefore, the metabolic changes associated with placental insufficiency are dependent on placental oxygen supply and cannot be alleviated by removing just the nutrient deprivation.

## 4. Skeletal Muscle Developmental Adaptations to IUGR Conditions

**Figure 1:**
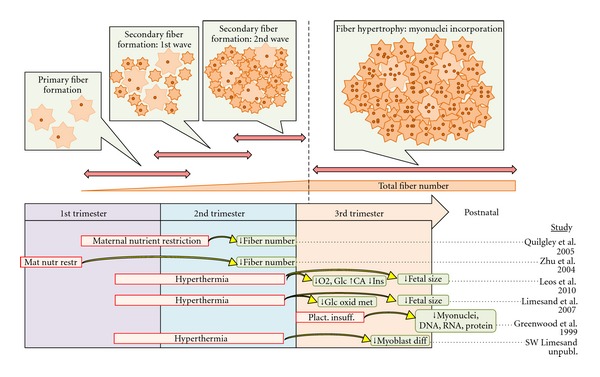
The stages of skeletal muscle formation relative to gestational age are depicted by the horizontal arrows and schematic diagrams (fascicular cross-sections) for the developmental process. The vertical dashed line represents the completion of myogenesis (new fiber formation) and onset of hypertrophic fiber growth. The timing, duration, and type of nutritional insult (red boxes) reported in various studies are presented below the gestational timeline, along with the fetal consequences (blue boxes).

The trajectory of skeletal muscle development and growth is slowed in IUGR fetuses. Ultrasonic measurements of IUGR fetuses show that muscle mass is reduced [51, 52], and animal studies show that nutrient restriction impairs fiber formation [53, 54]. Muscle fiber numbers, size, and metabolic phenotypes develop at distinct fetal stages and thus these aspects of muscle formation and growth are affected differently depending upon the timing of the fetal insult (Figure 1). Fiber numbers are determined by myogenesis (formation of new fibers), which occurs in 3 distinct phases and is completed early in the third trimester [55, 56]. Primary myotubes are generated from the fusion of progenitor cells midway through the first trimester, creating the scaffold around which smaller, secondary myotubes form near the end of the first trimester. A final wave of secondary (sometimes called tertiary) myotubes fills in the spaces not already occupied by existing fibers and completes myogenesis early in the third trimester. Nutritional insults during early or mid-gestation interfere with myotube formation and reduce fiber density in skeletal muscle. For example, maternal nutrient restriction between the mid-first and mid-second trimester in sheep lowers the number of secondary fibers per fasciculi in the fetal longissimus dorsi muscle [57]. In pregnant ewes recovering from malnourishment at peri-conception, secondary fiber density was also lower in the fetal semitendinosus muscle [58]. Although IUGR can result from maternal nutrient restriction during early gestation, placental insufficiency does not cause fetal hypoxemia and hypoglycemia until later stages of gestation, most likely after myogenesis is complete [53, 54]. As a result, placental insufficiency would reduce muscle mass by impairing fiber growth to a greater extent than total fiber number.

After myogenesis, muscle growth continues via fiber hypertrophy and requires myoblast incorporation to increase genomic DNA content [59–65]. Myonuclei incorporation precedes protein accumulation, and the size of a muscle fiber is dependent on DNA content [59–63]. Because muscle fiber myonuclei are postmitotic, DNA accumulation depends on incorporation of new nuclei from myoblasts [66]. In fact, 50–99% of total skeletal muscle DNA content accumulates postnatally [60]. In fetal sheep with placental insufficiency, skeletal muscle fibers contain fewer myonuclei than fibers from control fetuses, resulting in 33% less DNA, 40% less RNA, and 76% less protein per fiber [53, 54]. Human fetuses diagnosed as IUGR also have reduced skeletal muscle DNA content in late gestation but have normal protein-to-DNA ratios [67]. Our preliminary evidence indicates that myogenic cell populations are smaller in IUGR fetal skeletal muscle and that myoblasts isolated from IUGR fetal sheep may proliferate and differentiate at slower rates than those isolated from control fetuses (Yates, Limesand, and Rhoads, unpublished). This scenario would indicate that lower myonuclei content is a major limiting factor in IUGR skeletal muscle fiber growth and that IUGR myoblasts are impaired.

Histological measurements reveal a smaller proportion of oxidative-to-glycolytic muscle fibers in some skeletal muscles, which is another mechanism by which fetal developmental adaptations reduce muscle oxidative metabolism. In the ovine tibialis cranialis, newly forming secondary fibers express myosin-heavy chains for type II (glycolytic) fibers exclusively, but under normal conditions, ~60% of these fibers stain positive for type I (oxidative) myosin-heavy chains by the start of the third trimester [56]. The fiber-type ratio continues to shift toward oxidative fibers until a few weeks after birth [54, 68]. Together, these data reveal a multifaceted defect in IUGR skeletal muscle growth, which manifests in myoblast developmental programming that lowers myonuclei content and alters fiber phenotypes, thus preventing normal metabolic regulation.

## 5. Adrenergic Intervention: Catecholamines Change the Regulatory Signals

**Figure 2:**
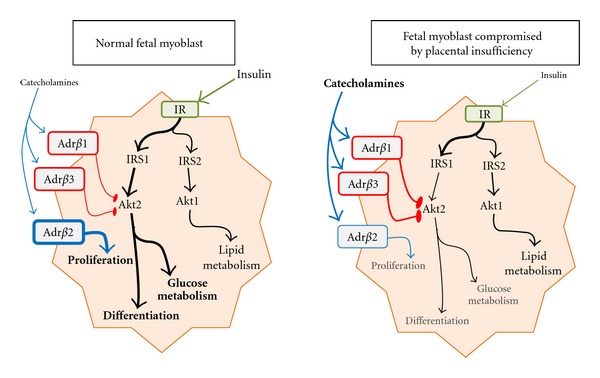
Impact of placental insufficiency on endocrine responsiveness in fetal myoblasts and myofibers. Adrenergic activity increases due to greater circulating catecholamines. Adrenergic receptor *β* subtype-specific desensitization results in a greater proportion of signaling through Adr*β*1 and Adr*β*3 because Adr*β*2 expression is reduced. Insulin signaling is reduced due to adrenergic suppression of insulin secretion in pancreatic *β*-cells and by muscle adrenergic signaling that negatively influences the insulin-Akt2 intercellular signaling pathway. These developmental adaptations reduce rates of myoblast proliferation and differentiation as well as glucose metabolism in skeletal muscle.

Adaptations in skeletal muscle growth and metabolism appear to be facilitated by chronic exposure to circulating catecholamines (Figure 2). In fact, intravenous infusion of norepinephrine or epinephrine for 8 days reduces plasma insulin and blood CO_2_, increases plasma lactate, and slows hindlimb muscle growth rate in otherwise uncompromised fetal sheep [69]. Catecholamines affect skeletal muscle directly by selectively impairing insulin signaling and indirectly by suppressing insulin secretion from pancreatic *β* cells [70, 71]. Under normal conditions, insulin regulates muscle metabolism by stimulating glucose uptake, glycogenesis, glucose oxidation, and protein synthesis via the Akt2 and MAPK-Erk1,2 signaling pathways [72–74] and by stimulating lipid metabolism via Akt1 [73]. Insulin also promotes myoblast proliferation and differentiation [75–77] by activating Akt2 via IRS1 [73, 77–79], and increases protein synthesis in fetal skeletal muscle [80, 81] and in myotubes derived from isolated fetal myoblasts [82]. However, placental insufficiency in fetal sheep reduces plasma insulin by 78% [20, 26, 69, 83] and skeletal muscle Akt2 content by 40% [48]. Furthermore, in adult rats chronically infused with epinephrine, insulin administration is less effective in stimulating IRS1 tyrosine phosphorylation, IRS1 complex with PI3K and SHP2, and Akt phosphorylation in skeletal muscle [84]. In adult humans, infusion of dobutamine (Adr*β*1 agonist) acutely reduces glucose oxidation rates and increases lipid oxidation rates in skeletal muscle [85]. Salbutamol (Adr*β*2 agonist) has no effect on glucose oxidation rates but slightly increases lipid oxidation [85]. Furthermore, catecholamines activate hormone-sensitive lipase to release fatty acids from fat stores [86, 87], which may help replace glucose as a metabolic substrate in muscle (Akt1 expression is not altered by catecholamines [48]). 

One major developmental adaptation in response to chronic catecholamine exposure is modified adrenergic signaling via alteration of Adr*β* expression. Findings in other tissues show that Adr*β*1, Adr*β*2, and Adr*β*3 have subtype-specific effects on insulin signaling. In adipocytes, Adr*β*1 and Adr*β*3 stimulation reduces insulin signaling by uncoupling IRS1 phosphorylation [88, 89] and Adr*β*1 suppresses insulin activation of Akt in cardiac muscle [90]. Conversely, Adr*β*2 amplifies insulin activation of MAPK-Erk1,2 in ovarian cells [91] and has been shown to stimulate myoblast proliferation directly in chicks and mice [92, 93]. However, we have found that expression of Adr*β*2 is reduced in myoblasts isolated from IUGR sheep fetuses (Table 1; Limesand and Yates, unpublished findings), meaning that adrenergic enhancement of insulin signaling is reduced. Meanwhile, myoblast Adr*β*1 and Adr*β*3, which inhibit insulin-stimulated proliferation and differentiation, are expressed normally. Likewise, Adr*β*2 mRNA expression is reduced in hindlimb skeletal muscle of IUGR fetal sheep and in those administered 7-day norepinephrine infusions, but Adr*β*1 and Adr*β*3 expression remain normal (SW Limesand and X Chen, unpublished data). The end result is a greater inhibitory effect on skeletal muscle insulin signaling which, along with reduced insulin secretion, would impair myoblast proliferation and incorporation into muscle fibers and insulin-driven glucose metabolism. Furthermore, skeletal muscle Adr*β*2 continues to be reduced in placental insufficiency-compromised lambs at one month of age, showing that the adaptive Adr*β* profile may be a contributing factor in postnatal metabolic disorders.

## 6. Fetal Adaptations Persist in Postnatal Life

Hypoglycemia and hypoxemia are alleviated by birth, but the thrifty metabolic adaptations persist into postnatal life [4, 5]. Children born with SGA have less skeletal muscle mass as infants and skeletal muscle mass grows at a slower rate through four years of age compared to their AGA counterparts [94–96]. Arm muscle size is reduced in infants at birth and at 3, 6, and 9 months of age [97] and upper-arm circumference and muscle area is less at 8 years of age [98]. Similarly, IUGR lambs have substantially reduced weight and protein content in the semitendinosus muscles at birth [53, 99], and daily protein accretion over the first few months of life is slowed [53]. As adults, SGA-born individuals have less lean muscle, greater fat-to-muscle ratios [100–103], and reduced muscle strength [102, 104]. Abdominal and leg muscle mass is reduced in otherwise healthy men at 19 and 22 years of age [105], and total lean muscle is lower at 50, 68, and 70 years of age [103, 106, 107]. In lambs and piglets, IUGR also impairs perinatal development of the vascular architecture [68, 108]. This may reflect an inability of myocytes to stimulate angiogenesis [109, 110] and is likely the origin of altered perfusion characteristics associated with metabolic syndrome, including vascular resistance, reduced responsiveness to adrenergic regulation, and endothelial dysfunction [111]. After birth, myoblasts form solely from the populations of quiescent satellite cells that develop along the basal lamina of muscle fibers [54, 112]. These populations, which control lifetime muscle growth and repair, accrue during fetal development and are subjected to IUGR conditions. Thus, the impairment of myoblast proliferation and differentiation responsible for slowing fetal skeletal muscle growth would also explain slower muscle growth rates in children and reduced lean mass in adults.

The thrifty metabolic phenotype that develops in utero also persists after birth. At 12 years of age, SGA-born children exhibit similar basal metabolic rates compared to AGA-born counterparts, but a smaller fraction of energy production is due to glucose oxidation and a larger fraction is from lipid oxidation [113]. Persistence of limited glucose oxidation rates in IUGR skeletal muscle can be associated with a combination of factors. First, less total lean muscle mass requires less energy. This scenario explains lower rates of systemic glucose oxidation but does not explain reduced muscle-specific glucose uptake [113, 114]. Dulloo [115, 116] postulates a second factor for reduced skeletal muscle glucose oxidation: glucose is preferentially redistributed to adipose tissues to replenish depleted fat stores. This “glucose redistribution hypothesis” has been applied to the perinatal period after IUGR as well as recovery from prolonged nutrient restriction at older ages [117]. However, SGA-born individuals continue to exhibit thrifty glucose metabolism throughout their lives, well after fat reserves are replenished, which indicates that the timing of the insult is important for persistence of the metabolic phenotype. Evidence for the permanence of developmental adaptations includes decreased oxidative-to-glycolytic fiber proportions in 8-month-old sheep exposed to fetal nutrient restriction and in mature pigs classified as runts (much smaller than littermates) at birth [45, 118]. Skeletal muscle biopsies from young-adult men born SGA reveal reduced insulin-signaling enzymes (e.g., PI3K, p85*α*, p110*β*, PKC*ζ*, Glut4) despite normal insulin receptor content [119]. In rats, insulin signaling via Akt is reduced in offspring from dams exposed to a hypoxic or malnourished environment during pregnancy [120]. Together, these studies indicate that the sustained response is not completed after adipose stores are replenish but is rather a product of a new nutrient utilization set-point established by fetal developmental programming to IUGR conditions. This phenomenon was described by Hales and Barker [4, 5] as “metabolic dysregulation,” but the connotation of a disorder may only apply because these individuals are subjected to a lifetime of diets that exceed their nutritional requirements.

## 7. Summary

Placental insufficiency results in conditions that restrict fetal skeletal muscle development and growth by reducing the capacity of the myofiber to maintain glucose homeostasis. Altered adrenergic receptor expression profiles in myoblasts and skeletal muscle of IUGR sheep fetuses indicate that slower growth rates and thrifty metabolism are the result of fetal adaptations to chronic catecholamine exposure in utero. As the proportion of Adr*β*2 to Adr*β*1 declines in IUGR skeletal muscle, adrenergic regulation promotes insulin resistance, reduced myoblast incorporation, less fiber hypertrophy, and lower rates of glucose oxidation. Developmental programming of skeletal muscle adrenergic receptors in utero helps explain metabolic and endocrine differences in IUGR offspring as well, and the impact on metabolism may result in differential nutrient utilization and requirements.

## Figures and Tables

**Table 1:** Adrenergic receptor *β* (Adr*β*) mRNA expression determined by quantitative PCR in placental insufficiency-induced IUGR^1^ and norepinephrine-infused^2^ sheep fetuses relative to control fetuses.

Treatment	Age at necropsy	Tissue	Adr*β*		
			Adr*β*1	Adr*β*2	Adr*β*3
PI-IUGR	Fetus, 134 dGA	Myoblasts^3^	↑28%	↓25%	↑800%
	Fetus, 134 dGA	Skeletal muscle^4^	NC	↓64%	NC
	Neonate, 28 days	Skeletal muscle^4^	—	↓44%	—
NE-Infused	Fetus, 140 dGA	Skeletal muscle^4^	NC	↓47%	NC

^1^Hyperthermia from 40 to 95 days of gestation (term ~145 days).

^2^Intravenous norepinephrine (NE) infusions from 130 to 137 days of gestational age.

^3^Isolated from hindlimb skeletal muscles. *n* = 3/treatment.

^4^Pooled semitendinosus and biceps femoris. *n* = 6/treatment.

NC: no change; ↑: increased relative to controls; ↓: decreased relative to controls. Constitutive control was s15 for all samples.

## References

[1] Saleem T, Sajjad N, Fatima S, Habib N, Ali S, Qadir M (2011). Intrauterine growth retardation-small events, big consequences. *Italian Journal of Pediatrics*.

[2] Berghella V (2007). Prevention of recurrent fetal growth restriction. *Obstetrics and Gynecology*.

[3] Alisi A, Panera N, Agostoni C, Nobili V (2011). Intrauterine growth retardation and nonalcoholic fatty liver disease in children. *International Journal of Endocrinology*.

[4] Hales CN, Barker DJP (1992). Type 2 (non-insulin-dependent) diabetes mellitus: the thrifty phenotype hypothesis. *Diabetologia*.

[5] Hales CN, Barker DJP (2001). The thrifty phenotype hypothesis. *British Medical Bulletin*.

[6] Flanagan DE, Moore VM, Godsland IF, Cockington RA, Robinson JS, Phillips DI (2000). Fetal growth and the physiological control of glucose tolerance in adults: a minimal model analysis. *American Journal of Physiology-Endocrinology and Metabolism*.

[7] Mericq V, Ong KK, Bazaes R (2005). Longitudinal changes in insulin sensitivity and secretion from birth to age three years in small- and appropriate-for-gestational-age children. *Diabetologia*.

[8] Ong KKL, Ahmed ML, Dunger DB, Emmett PM, Preece MA (2000). Association between postnatal catch-up growth and obesity in childhood: prospective cohort study. *British Medical Journal*.

[9] Vickers MH, Breier BH, Cutfield WS, Hofman PL, Gluckman PD (2000). Fetal origins of hyperphagia, obesity, and hypertension and postnatal amplification by hypercaloric nutrition. *American Journal of Physiology-Endocrinology and Metabolism*.

[10] Barker DJP, Hales CN, Fall CHD, Osmond C, Phipps K, Clark PMS (1993). Type 2 (non-insulin-dependent) diabetes mellitus, hypertension and hyperlipidaemia (syndrome X): relation to reduced fetal growth. *Diabetologia*.

[11] Ravelli ACJ, Van Der Meulen JHP, Michels RPJ (1998). Glucose tolerance in adults after prenatal exposure to famine. *The Lancet*.

[12] Newsome CA, Shiell AW, Fall CHD, Phillips DIW, Shier R, Law CM (2003). Is birth weight related to later glucose and insulin metabolism?—A systematic review. *Diabetic Medicine*.

[13] Gatford KL, Simmons RA, De Blasio MJ, Robinson JS, Owens JA (2010). Review: placental programming of postnatal diabetes and impaired insulin action after IUGR. *Placenta*.

[14] Hay WW, Thureen P (2010). Protein for preterm infants: how much is needed? How much is enough? How much is too much?. *Pediatrics and Neonatology*.

[15] Ghidini A (1996). Idiopathic fetal growth restriction: a pathophysiologic approach. *Obstetrical and Gynecological Survey*.

[16] Okamura K, Watanabe T, Tanigawara S (1990). Catecholamine levels and their correlation to blood gases in umbilical venous blood obtained by cordocentesis. *Fetal Diagnosis and Therapy*.

[17] Harwell CM, Padbury JF, Anand RS (1990). Fetal catecholamine responses to maternal hypoglycemia. *American Journal of Physiology-Regulatory Integrative and Comparative Physiology*.

[18] Phillippe M, Kitzmiller JL (1981). The fetal and maternal catecholamine response to insulin-induced hypoglycemia in the rat. *American Journal of Obstetrics and Gynecology*.

[19] Cohen WR, Piasecki GJ, Cohn HE, Susa JB, Jackson BT (1991). Sympathoadrenal responses during hypoglycemia, hyperinsulinemia, and hypoxemia in the ovine fetus. *American Journal of Physiology-Endocrinology and Metabolism*.

[20] Jackson BT, Cohn HE, Morrison SH, Baker RM, Piasecki GJ (1993). Hypoxia-induced sympathetic inhibition of the fetal plasma insulin response to hyperglycemia. *Diabetes*.

[21] Cheung CY (1990). Fetal adrenal medulla catecholamine response to hypoxia-direct and neural components. *American Journal of Physiology-Regulatory Integrative and Comparative Physiology*.

[22] Rychkov GY, Adams MB, McMillen IC, Roberts ML (1998). Oxygen sensing mechanisms are present in the chromaffin cells of the sheep adrenal medulla before birth. *Journal of Physiology*.

[23] Greenough A, Nicolaides KH, Lagercrantz H (1990). Human fetal sympathoadrenal responsiveness. *Early Human Development*.

[24] Lagercrantz H, Sjoquist B, Bremme K (1980). Catecholamine metabolites in amniotic fluid as indicators of intrauterine stress. *American Journal of Obstetrics and Gynecology*.

[25] Hiraoka T, Kudo T, Kishimoto Y (1991). Catecholamines in experimentally growth-retarded rat fetus. *Asia-Oceania Journal of Obstetrics and Gynaecology*.

[26] Leos RA, Anderson MJ, Chen X, Pugmire J, Anderson KA, Limesand SW (2010). Chronic exposure to elevated norepinephrine suppresses insulin secretion in fetal sheep with placental insufficiency and intrauterine growth restriction. *American Journal of Physiology-Endocrinology and Metabolism*.

[27] Limesand SW, Rozance PJ, Zerbe GO, Hutton JC, Hay WW (2006). Attenuated insulin release and storage in fetal sheep pancreatic islets with intrauterine growth restriction. *Endocrinology*.

[28] Jones CT, Robinson JS (1983). Studies on experimental growth retardation in sheep. Plasma catecholamines in fetuses with small placenta. *Journal of Developmental Physiology*.

[29] Altan VM, Arioglu E, Guner S, Ozcelikay AT (2007). The influence of diabetes on cardiac *β*-adrenoceptor subtypes. *Heart Failure Reviews*.

[30] Schaak S, Mialet-Perez J, Flordellis C, Paris H (2007). Genetic variation of human adrenergic receptors: from molecular and functional properties to clinical and pharmacogenetic implications. *Current Topics in Medicinal Chemistry*.

[31] Dohlman HG, Thorner J, Caron MG, Lefkowitz RJ (1991). Model systems for the study of seven-transmembrane-segment receptors. *Annual Review of Biochemistry*.

[32] Goodlin RC, Lowe EW (1974). A functional umbilical cord occlusion heart rate pattern. The significance of overshoot. *Obstetrics and Gynecology*.

[33] Robinson JS, Jones CT, Thorburn GD (1977). The effects of hypoxaemia in fetal sheep. *Journal of Clinical Pathology*.

[34] Hay WW, Walker W, Watkins J, Duggan C (2003). Nutrition and development of the fetus: carbohydrate and lipid metabolism. *Nutrition in Pediatrics (Basic Science and Clinical Applications)*.

[35] Jackson BT, Piasecki GJ, Cohn HE, Cohen WR (2000). Control of fetal insulin secretion. *American Journal of Physiology-Regulatory Integrative and Comparative Physiology*.

[36] Sperling MA, Christensen RA, Ganguli S, Anand R (1980). Adrenergic modulation of pancreatic hormone secretion in utero: studies in fetal sheep. *Pediatric Research*.

[37] Yates DT, Green AS, Limesand SW (2011). Catecholamines mediate multiple fetal adaptations during placental insufficiency that contribute to intrauterine growth restriction: lessons from hyperthermic sheep. *Journal of Pregnancy*.

[38] Lane RH, Chandorkar AK, Flozak AS, Simmons RA (1998). Intrauterine growth retardation alters mitochondrial gene expression and function in fetal and juvenile rat skeletal muscle. *Pediatric Research*.

[39] Limesand SW, Rozance PJ, Brown LD, Hay WW (2009). Effects of chronic hypoglycemia and euglycemic correction on lysine metabolism in fetal sheep. *American Journal of Physiology-Endocrinology and Metabolism*.

[40] Van Veen LCP, Teng C, Hay WW (1987). Leucine disposal and oxidation rates in the fetal lamb. *Metabolism*.

[41] Carver TD, Quick AA, Teng CC, Pike AW, Fennessey PV, Hay WW (1997). Leucine metabolism in chronically hypoglycemic hypoinsulinemic growth- restricted fetal sheep. *American Journal of Physiology-Endocrinology and Metabolism*.

[42] Galan HL, Anthony RV, Rigano S (2005). Fetal hypertension and abnormal Doppler velocimetry in an ovine model of intrauterine growth restriction. *American Journal of Obstetrics and Gynecology*.

[43] Regnault TRH, de Vrijer B, Galan HL, Wilkening RB, Battaglia FC, Meschia G (2007). Development and mechanisms of fetal hypoxia in severe fetal growth restriction. *Placenta*.

[44] Chen X, Fahy AL, Green AS, Anderson MJ, Rhoads RP, Limesand SW (2010). *β*2-Adrenergic receptor desensitization in perirenal adipose tissue in fetuses and lambs with placental insufficiency-induced intrauterine growth restriction. *Journal of Physiology*.

[45] Zhu MJ, Ford SP, Means WJ, Hess BW, Nathanielsz PW, Du M (2006). Maternal nutrient restriction affects properties of skeletal muscle in offspring. *Journal of Physiology*.

[46] Germani D, Puglianiello A, Cianfarani S (2008). Uteroplacental insufficiency down regulates insulin receptor and affects expression of key enzymes of long-chain fatty acid (LCFA) metabolism in skeletal muscle at birth. *Cardiovascular Diabetology*.

[47] Limesand SW, Rozance PJ, Smith D, Hay WW (2007). Increased insulin sensitivity and maintenance of glucose utilization rates in fetal sheep with placental insufficiency and intrauterine growth restriction. *American Journal of Physiology-Endocrinology and Metabolism*.

[48] Thorn SR, Regnault TRH, Brown LD (2009). Intrauterine growth restriction increases fetal hepatic gluconeogenic capacity and reduces messenger ribonucleic acid translation initiation and nutrient sensing in fetal liver and skeletal muscle. *Endocrinology*.

[49] Limesand SW, Hay WW (2003). Adaptation of ovine fetal pancreatic insulin secretion to chronic hypoglycaemia and euglycaemic correction. *Journal of Physiology*.

[50] Rozance PJ, Limesand SW, Barry JS, Brown LD, Hay WW (2009). Glucose replacement to euglycemia causes hypoxia, acidosis, and decreased insulin secretion in fetal sheep with intrauterine growth restriction. *Pediatric Research*.

[51] Padoan A, Rigano S, Ferrazzi E, Beaty BL, Battaglia FC, Galan HL (2004). Differences in fat and lean mass proportions in normal and growth-restricted fetuses. *American Journal of Obstetrics and Gynecology*.

[52] Larciprete G, Valensise H, Di Pierro G (2005). Intrauterine growth restriction and fetal body composition. *Ultrasound in Obstetrics and Gynecology*.

[53] Greenwood PL, Hunt AS, Hermanson JW, Bell AW (2000). Effects of birth weight and postnatal nutrition on neonatal sheep: II. Skeletal muscle growth and development. *Journal of Animal Science*.

[54] Greenwood PL, Slepetis RM, Hermanson JW, Bell AW (1999). Intrauterine growth retardation is associated with reduced cell cycle activity, but not myofibre number, in ovine fetal muscle. *Reproduction, Fertility and Development*.

[55] Wilson SJ, McEwan JC, Sheard PW, Harris AJ (1992). Early stages of myogenesis in a large mammal: formation of successive generations of myotubes in sheep tibialis cranialis muscle. *Journal of Muscle Research and Cell Motility*.

[56] Maier A, McEwan JC, Dodds KG, Fischman DA, Fitzsimons RB, Harris AJ (1992). Myosin heavy chain composition of single fibres and their origins and distribution in developing fascicles of sheep tibialis cranialis muscles. *Journal of Muscle Research and Cell Motility*.

[57] Zhu MJ, Ford SP, Nathanielsz PW, Du M (2004). Effect of maternal nutrient restriction in sheep on the development of fetal skeletal muscle. *Biology of Reproduction*.

[58] Quigley SP, Kleemann DO, Kakar MA (2005). Myogenesis in sheep is altered by maternal feed intake during the peri-conception period. *Animal Reproduction Science*.

[59] Winick M, Noble A (1965). Quantitative changes in DNA, RNA, and protein during prenatal and postnatal growth in the rat. *Developmental Biology*.

[60] Allen RE, Merkel RA, Young RB (1979). Cellular aspects of muscle growth: myogenic cell proliferation. *Journal of Animal Science*.

[61] Swatland HJ (1977). Accumulation of myofiber nuclei in pigs with normal and arrested development. *Journal of Animal Science*.

[62] Trenkle A, DeWitt DL, Topel DG (1978). Influence of age, nutrition and genotype on carcass traits and cellular development of the M. Longissimus of cattle. *Journal of Animal Science*.

[63] Harbison SA, Goll DE, Parrish FC (1976). Muscle growth in two genetically different lines of swine. *Growth*.

[64] Ten Broek RW, Grefte S, Von Den Hoff JW (2010). Regulatory factors and cell populations involved in skeletal muscle regeneration. *Journal of Cellular Physiology*.

[65] Davis TA, Fiorotto ML (2009). Regulation of muscle growth in neonates. *Current Opinion in Clinical Nutrition and Metabolic Care*.

[66] Moss FP, Leblond CP (1971). Satellite cells as the source of nuclei in muscles of growing rats. *Anatomical Record*.

[67] Widdowson EM, Crabb DE, Milner RD (1972). Cellular development of some human organs before birth. *Archives of Disease in Childhood*.

[68] Costello PM, Rowlerson A, Astaman NA (2008). Peri-implantation and late gestation maternal undernutrition differentially affect fetal sheep skeletal muscle development. *Journal of Physiology*.

[69] Bassett JM, Hanson C (1998). Catecholamines inhibit growth in fetal sheep in the absence of hypoxemia. *American Journal of Physiology-Regulatory Integrative and Comparative Physiology*.

[70] Milley JR (1997). Ovine fetal metabolism during norepinephrine infusion. *American Journal of Physiology-Endocrinology and Metabolism*.

[71] Bassett JM, Hanson C (2000). Prevention of hypoinsulinemia modifies catecholamine effects in fetal sheep. *American Journal of Physiology-Regulatory Integrative and Comparative Physiology*.

[72] Shepherd PR, Withers DJ, Siddle K (1998). Phosphoinositide 3-kinase: the key switch mechanism in insulin signalling. *Biochemical Journal*.

[73] Bouzakri K, Karlsson HKR, Vestergaard H, Madsbad S, Christiansen E, Zierath JR (2006). IRS-1 serine phosphorylation and insulin resistance in skeletal muscle from pancreas transplant recipients. *Diabetes*.

[74] Hay WW, DiGiacomo JE, Meznarich HK, Hirst K, Zerbe G (1989). Effects of glucose and insulin on fetal glucose oxidation and oxygen consumption. *American Journal of Physiology-Endocrinology and Metabolism*.

[75] Allen RE, Luiten LS, Dodson MV (1985). Effect of insulin and linoleic acid on satellite cell differentiation. *Journal of Animal Science*.

[76] Castillo J, Codina M, Martinez ML, Navarro I, Gutierrez J (2004). Metabolic and mitogenic effects of IGF-I and insulin on muscle cells of rainbow trout. *American Journal of Physiology-Regulatory, Integrative and Comparative Physiology*.

[77] Vandromme M, Rochat A, Meier R (2001). Protein Kinase B *β*/Akt2 Plays a Specific Role in Muscle Differentiation. *Journal of Biological Chemistry*.

[78] Sumitani S, Goya K, Testa JR, Kouhara H, Kasayama S (2002). Akt1 and Akt2 differently regulate muscle creatine kinase and myogenin gene transcription in insulin-induced differentiation of C2C12 myoblasts. *Endocrinology*.

[79] Calera MR, Pilch PF (1998). Induction of Akt-2 correlates with differentiation in Sol8 muscle cells. *Biochemical and Biophysical Research Communications*.

[80] Brown LD, Rozance PJ, Barry JS, Friedman JE, Hay WW (2009). Insulin is required for amino acid stimulation of dual pathways for translational control in skeletal muscle in the late-gestation ovine fetus. *American Journal of Physiology-Endocrinology and Metabolism*.

[81] Brown LD, Hay WW (2006). Effect of hyperinsulinemia on amino acid utilization and oxidation independent of glucose metabolism in the ovine fetus. *American Journal of Physiology-Endocrinology and Metabolism*.

[82] Harper JMM, Soar JB, Buttery PJ (1987). Changes in protein metabolism of ovine primary muscle cultures on treatment with growth hormone, insulin, insulin-like growth factor I or epidermal growth factor. *Journal of Endocrinology*.

[83] Sperling MA, Ganguli S, Leslie N, Landt K (1984). Fetal-perinatal catecholamine secretion: role in perinatal glucose homeostasis. *The American Journal of Physiology*.

[84] Lima MHM, Ueno M, Thirone ACP, Rocha EM, Carvalho CRO, Saad MJA (2002). Regulation of IRS-1/SHP2 interaction and AKT phosphorylation in animal models of insulin resistance. *Endocrine*.

[85] Hoeks J, Van Baak MA, Hesselink MKC (2003). Effect of *β*1- and *β*2-adrenergic stimulation on energy expenditure, substrate oxidation, and UCP3 expression in humans. *American Journal of Physiology-Endocrinology and Metabolism*.

[86] Carmen GY, Víctor SM (2006). Signalling mechanisms regulating lipolysis. *Cellular Signalling*.

[87] Tansey JT, Sztalryd C, Hlavin EM, Kimmel AR, Londos C (2004). The central role of perilipin A in lipid metabolism and adipocyte lipolysis. *IUBMB Life*.

[88] Jost MM, Jost P, Klein J, Klein HH (2005). The *β*3-adrenergic agonist CL316,243 inhibits insulin signaling but not glucose uptake in primary human adipocytes. *Experimental and Clinical Endocrinology and Diabetes*.

[89] Jost P, Fasshauer M, Kahn CR (2002). Atypical *β*-adrenergic effects on insulin signaling and action in *β*3-adrenoceptor-deficient brown adipocytes. *American Journal of Physiology-Endocrinology and Metabolism*.

[90] Morisco C, Condorelli G, Trimarco V (2005). Akt mediates the cross-talk between *β*-adrenergic and insulin receptors in neonatal cardiomyocytes. *Circulation Research*.

[91] Wang H, Doronin S, Malbon CC (2000). Insulin activation of mitogen-activated protein kinases Erk1,2 is amplified via *β*-adrenergic receptor expression and requires the integrity of the Tyr350 of the receptor. *Journal of Biological Chemistry*.

[92] Grant AL, Helferich WG, Merkel RA, Bergen WG (1990). Effects of phenethanolamines and propranolol on the proliferation of cultured chick breast muscle satellite cells. *Journal of Animal Science*.

[93] Roberts P, McGeachie JK (1996). Long-term isoprenaline administration and its effect on the revascularisation and regeneration of skeletal muscle transplants in mice. *Journal of Anatomy*.

[94] Lapillonne A, Braillon P, Claris O, Chatelain PG, Delmas PD, Salle BL (1997). Body composition in appropriate and in small for gestational age infants. *Acta Paediatrica*.

[95] Hediger ML, Overpeck MD, Kuczmarski RJ, McGlynn A, Maurer KR, Davis WW (1998). Muscularity and fatness of infants and young children born small- or large-for-gestational-age. *Pediatrics*.

[96] Ibáñez L, Ong K, Dunger DB, De Zegher F (2006). Early development of adiposity and insulin resistance after catch-up weight gain in small-for-gestational-age children. *Journal of Clinical Endocrinology and Metabolism*.

[97] Bhatia BD, Agarwal KN, Jain NP (1983). Muscle mass of intrauterine growth retarded babies in first nine months of life. *Indian Pediatrics*.

[98] Lima MC, Dantas HF, Amorim RJM, Lira PIC (2011). Does fetal growth restriction influence body composition at school age?. *Jornal de Pediatria*.

[99] Greenwood PL, Hunt AS, Hermanson JW, Bell AW (1998). Effects of birth weight and postnatal nutrition on neonatal sheep: I. Body growth and composition, and some aspects of energetic efficiency. *Journal of Animal Science*.

[100] Sayer AA, Syddall HE, Dennison EM (2004). Birth weight, weight at 1 y of age, and body composition in older men: findings from the Hertfordshire Cohort Study. *The American Journal of Clinical Nutrition*.

[101] Gale CR, Martyn CN, Kellingray S, Eastell R, Cooper C (2001). Intrauterine programming of adult body composition. *Journal of Clinical Endocrinology and Metabolism*.

[102] Yliharsila H, Kajantie E, Osmond C, Forsén T, Barker DJP, Eriksson JG (2007). Birth size, adult body composition and muscle strength in later life. *International Journal of Obesity*.

[103] Kensara OA, Wootton SA, Phillips DI, Patel M, Jackson AA, Elia M (2005). Fetal programming of body composition: relation between birth weight and body composition measured with dual-energy X-ray absorptiometry and anthropometric methods in older Englishmen. *American Journal of Clinical Nutrition*.

[104] Inskip HM, Godfrey KM, Martin HJ, Simmonds SJ, Cooper C, Sayer AA (2007). and the Southampton Women’s survey study G. size at birth and its relation to muscle strength in young adult women. *Journal of Internal Medicine*.

[105] Rasmussen EL, Malis C, Jensen CB (2005). Altered fat tissue distribution in young adult men who had low birth weight. *Diabetes Care*.

[106] Phillips DIW (1995). Relation of fetal growth to adult muscle mass and glucose tolerance. *Diabetic Medicine*.

[107] Eriksson J, Forsén T, Tuomilehto J, Osmond C, Barker D (2002). Size at birth, fat-free mass and resting metabolic rate in adult life. *Hormone and Metabolic Research*.

[108] Bauer R, Gedrange T, Bauer K, Walter B (2006). Intrauterine growth restriction induces increased capillary density and accelerated type I fiber maturation in newborn pig skeletal muscles. *Journal of Perinatal Medicine*.

[109] Chiristov C, Chrétien F, Abou-Khalil R (2007). Muscle satellite cells and endothelial cells: close neighbors and privileged partners. *Molecular Biology of the Cell*.

[110] Rhoads RP, Johnson RM, Rathbone CR (2009). Satellite cell-mediated angiogenesis in vitro coincides with a functional hypoxia-inducible factor pathway. *American Journal of Physiology-Cell Physiology*.

[111] Frisbee JC, Wu F, Goodwill AG, Butcher JT, Beard DA (2011). Spatial heterogeneity in skeletal muscle microvascular blood flow distribution is increased in the metabolic syndrome. *American Journal of Physiology-Regulatory Integrative and Comparative Physiology*.

[112] Messina G, Cossu G (2009). The origin of embryonic and fetal myoblasts: a role of Pax3 and Pax7. *Genes and Development*.

[113] Jornayvaz FR, Selz R, Tappy L, Theintz GE (2004). Metabolism of oral glucose in children born small for gestational age: evidence for an impaired whole body glucose oxidation. *Metabolism*.

[114] Hermann TS, Rask-Madsen C, Ihlemann N (2003). Normal insulin-stimulated endothelial function and impaired insulin-stimulated muscle glucose uptake in young adults with low birth weight. *Journal of Clinical Endocrinology and Metabolism*.

[115] Dulloo AG (2006). Regulation of fat storage via suppressed thermogenesis: a thrifty phenotype that predisposes individuals with catch-up growth to insulin resistance and obesity. *Hormone Research*.

[116] Dulloo AcG (2008). Thrifty energy metabolism in catch-up growth trajectories to insulin and leptin resistance. *Best Practice and Research in Clinical Endocrinology and Metabolism*.

[117] Cettour-Rose P, Samec S, Russell AP (2005). Redistribution of glucose from skeletal muscle to adipose tissue during catch-up fat: a link between catch-up growth and later metabolic syndrome. *Diabetes*.

[118] Powell SE, Aberle ED (1981). Skeletal muscle and adipose tissue cellularity in runt and normal birth weight swine. *Journal of Animal Science*.

[119] Ozanne SE, Jensen CB, Tingey KJ, Storgaard H, Madsbad S, Vaag AA (2005). Low birthweight is associated with specific changes in muscle insulin-signalling protein expression. *Diabetologia*.

[120] Camm EJ, Martin-Gronert MS, Wright NL, Hansell JA, Ozanne SE, Giussani DA (2011). Prenatal hypoxia independent of undernutrition promotes molecular markers of insulin resistance in adult offspring. *FASEB Journal*.

